# Time Trends in Adolescent School Absences and Associated Bullying Involvement Between 2000 and 2019: A Nationwide Study

**DOI:** 10.1007/s10578-023-01601-1

**Published:** 2023-08-26

**Authors:** K. Alanko, K. Melander, K. Ranta, J. Engblom, S. Kosola

**Affiliations:** 1 Faculty of Humanities, Psychology and Theology, Åbo Akademi University, Turku, Finland; 2 Tampere University Hospital, and University of Tampere, University of Helsinki, Helsinki, Finland; 3 Faculty of Social Sciences, Faculty of Medicine, Tampere University, University of Helsinki, Tampere, Helsinki, Finland; 4 Department of Mathematics and Statistics, School of Economics, University of Turku, University of Turku, Turku, Finland; 5 Pediatric Research Center, New Children’s Hospital, Helsinki University Hospital, University of Helsinki, Helsinki, Finland

**Keywords:** School absence, Illness absence, Truancy, Time trend, Bullying

## Abstract

Education is a central determinant of adolescent health. School absences and bullying involvement jeopardize wellbeing, mental health, and educational attainment. We analyzed time trends in school absenteeism over two decades and examined the association of absenteeism with bullying involvement.

We analyzed data from the nationwide School Health Promotion study, with self-reported data from Finnish middle school students in grades 8 and 9 (ages 14–17, N = 1 000 970). Questionnaires assessed frequency of illness absences (IA), truancy, frequency of bullying victimization, bullying perpetration, and involvement in both bullying perpetration and victimization. Frequent school absences were defined as occurring on more than 3 days during the prior month (2000–2015), or at least weekly (2017–2019).

Frequent IA increased from 12% to 2000 to 22% in 2015. In 2017–2019, frequent IA was reported by 3.5%. Frequent truancy declined from 9% to 2000 to 4% in 2015, and remained at 4% during 2017–2019. Bully victimization was reported at least weekly by 6.9%, perpetration by 5.4% and victimization-perpetration by 1.9% of participants in total. In a logistic regression model, every type of bullying involvement increased odds for both IA and truancy.

Since bullying involvement was associated with both IA and truancy, particular concern should be raised for adolescents involved in bullying, and for their social and educational functioning. The concurrent increase in IA and decrease in truancy may reflect destigmatization of mental health problems or other changes in reporting absenteeism.

## Introduction

School attendance is one of the key determinants of healthy development in children and adolescents [1, 2] School absenteeism, in contrast, is associated with poor academic performance, mental health problems such as depression, anxiety, and conduct disorders, as well as increased risk for substance use [2, 3]. Absenteeism also associates with impairments in social functioning, and prolonged absenteeism increases the risk of school dropout and even extreme social withdrawal [4]. Longitudinal studies suggest that prolonged absenteeism poses risks for low societal and vocational adaptation in the future [l]. The reasons for absenteeism are heterogenous and often complex [2, 3].

School absences are often divided into legitimate/excused and illegitimate/unexcused absences (also referred to as truancy). Most absences are legitimate [4]. A majority of them are due to common illnesses (illness absences, IA) verified by either a caregiver or a medical professional, while some reasons for school absence are approved by the caregivers and school (e.g., participation in family or sports events). Truancy refers to absences that neither the caregiver nor the school have approved [2]. Kearney proposed that absenteeism severity could be determined according to percentage of time absent from school, or more lately, according to the functional deficits imposed upon the child in different contexts: social, family, school or spare time [5].

Absence rates vary as a function of school system [6], culture [1, 5], operationalization, and informant [9]. In a cross-sectional study, 32–35% of 14-to-15-year-old Dutch students reported IA during the prior 30 days [10]. In Sweden, recurring IAs were reported by 9.5% of students in middle school [11]. In the Programme for International Student Assessment (PISA) report from 2019, 21% of 15-year-old students internationally, and 13% of students in Finland reported truancy at least once during the previous two weeks, boys more often than girls [8]. Severe truancy, i.e. more than three times during the previous two weeks, was reported by 2.5% of high school students in Finland [6].

Large population studies on temporal changes in the frequency of legitimate and illegitimate school absences are infrequent. A recent government report using register-data in Scotland found no temporal change in IA in secondary schools as rates were 4.3% in 2010, and 4.4% in 2019, reported as percentage of total annual school time [12]. However, truancy showed a slight increase from 2.1% to 2010 to 2.9% in 2019. In the 2019 PISA report, truancy increased by 1% from 2015 to 2018 internationally [8], yet in Finland a concurrent 23% decline in self-reported truancy occurred [8]. In the United States, self-reported truancy rates remained stable at approximately 11% among adolescents aged 12 to 17 between 2002 and 2014, with higher rates among females, older adolescents, and Hispanic adolescents [13].

Prior findings on the associations between school absences and bullying involvement are inconsistent: some studies reported a direct association [5, 11, 12] while others found no association [16]. Furthermore, many studies have focused on the association between truancy and bullying involvement but overlooked IA. Bullying victimization is associated with psychological, behavioral, social, and academic issues, and in some studies, truancy [15], [17] as well as absenteeism generally [8, 15, 18]. In a nationally representative sample of U.S. adolescents, bullying victims reported school absenteeism nearly four times as often as their non-bullied peers (15.5% vs. 4.1%) [12]. Truant adolescents are, however, also likely to manifest a range of behavior problems, such as bullying perpetration [16]. Bullying perpetration-victimization is associated with poor relationships with teachers and absenteeism [17, 18].

The present study aimed to examine temporal changes in the prevalence of IA and truancy among adolescents in a nationally representative sample gathered over a period of 20 years. The second aim was to study the associations of IA and truancy with bullying victimization, perpetration and victimization-perpetration.

## Methods

### Procedure

The study is based on a large, biennial survey, The School Health Promotion study (SHP), conducted by the Finnish Institute of Health and Welfare (THL). All students in grades 8 and 9 in Finland were invited to participate during a school day in the spring term. Participation was voluntary and anonymous. Prior to 2013, the study was implemented in Southern, Eastern and Northern Finland on even-numbered years and in Western and Central Finland on odd-numbered years. After 2013, data has been collected from the entire country biennially and students have had the possibility to respond electronically. Previously, and also as a complement after 2013, a paper version of the survey was provided. Annual response rates varied between 63 and 84%. In 2015, technical problems led to a lower response rate. The typical age for 8th and 9th graders is 13–16 years, therefore responses with age below 13 or over 17 were excluded as non-serious replies. The study was approved by the THL Working Group on Research Ethics.

### Demographic and Socioeconomic Background

Demographic variables in the present study are sex (as sex assigned at birth: male, female), grade (grade 8 or 9), and maternal educational level (1 = comprehensive school or equivalent, 2 = upper secondary school, high school or vocational education institution, 3 = occupational studies in addition to upper secondary school, high school or vocational education institution and, 4 = university, university of applied sciences or other higher education institution).

### Illness Absences and Truancy

Self-reported IA and truancy were measured each year. From 2000 to 2015, the following item was used: “During the past 30 days, how many days have you been absent from school due to truancy/illness/other reason?”. Response options were categorical: none, one day, 2–3 days, more than 3 days. In 2017 and 2019, the question read: “During this school year, how often have you experienced the following: being late, being absent without permission: skipping school, or being absent due to illness?”. The response options were: not at all, a few times in the year, every month, every week, daily or almost daily.

To facilitate analyses across the whole study period, frequency of IA was coded into three categories: infrequent (none or one day per month, or not at all or a few times in the year); moderate (2–3 days per month, and every month); or frequent (more than 3 days per month, or every week, daily or almost daily during the last school year). Truancy was also coded into three categories: infrequent (none and not at all) moderate (maximum 2–3 days; and a few times per year or every month) and frequent (more than 3 days and every week, daily or almost daily).

### Involvement in Bullying

Bullying involvement items were derived from a World Health Organization study on youth health [21], based on Olweus’ definition of bullying [22]. First, a definition of bullying was provided: “We say a student is being bullied when another student (or group of students), say or do nasty things to him or her. It is also bullying when a student is being teased repeatedly in a way she or he does not like. But it is not bullying when two students of about the same strength quarrel or fight.” Bully victimization was then measured with one item: “How often have you been bullied at school during this semester?”. Bully perpetration was measured with one item: “How often have you participated in bullying other students during this semester?”. Response options for both items were: several times a week, about once a week, less frequently, not at all.

Students who reported both victimization and perpetration at least once a week were coded as bully victim-perpetrators for the analyses of associations; however, this category was not included in the time trend analyses.

### Data Analyses

All analyses were conducted using SAS 9.4 software. Descriptive data are presented as frequencies, and cross-tabulations with chi-square statistics. In the Spearman correlational analyses, to analyse if the strength of the association between bullying and absence had changed over time, sex, grade, and maternal education level were included as covariates. We used cumulative logistic regression (LR), which yields estimated adjusted odds ratios (OR) with 95% confidence intervals (CI), to model the effects of sex, age, maternal education, study year, bullying involvement on IA or truancy.

## Results

The total sample size was 1 000 970, and mean age was 15.34 (*SD =* 0.62; Table 1). Sex and age distributions of participants were even.

**Table 1:** Descriptive statistics of the study population

		Total N		Any truancy		Any illness absences	
Year	N	% girls	% in grade 8	% of girls	% of boys	% of girls	% of boys
2000–2001	83,094	50.0	50.0	28.7	25.6	34.1	31.7
2002–2003	98,601	49.4	51.3	22.2	19.7	30.2	27.4
2004–2005	48,142	49.5	51.4	21.1	19.5	31.9	29.5
2006–2007	106,949	49.9	51.0	21.7	19.8	34.9	33.1
2008–2009	105,597	50.1	49.8	23.6	20.0	36.8	34.3
2010–2011	99,538	50.1	49.7	21.7	18.5	35.5	33.2
2012–2013	96,124	49.5	49.6	18.3	16.2	38.8	35.9
2014–2015	49,339	50.3	50.4	17.7	16.8	45.8	42.6
2016–2017	72,402	50.4	50.1	25.2	31.1	25.4	21.2
2018–2019	86,132	50.7	51.0	25.3	31.1	26.2	22.7

Note. Study population included students in grades 8 and 9. Above, the proportion of participants in grade 8 is presented. Any IA included answers other than a few times during the year (2017–2019) or 1 day during the prior 30 days (2000–2015). Any truancy included answers other than “none” and “not at all”

Over the entire study period, frequent IA was reported by 12% of students, moderate by 20%, and 32% of students reported infrequent/no IA (Table 2). Frequent and moderate IA were somewhat more common among girls over the entire study period (χ² = 807.15, p < 0.001). Eighteen per cent of adolescents reported some truancy, and among 4% truancy was frequent. Truancy in general was more common among girls, whereas frequent truancy was more common among boys (χ² = 496.01, p < 0.001).

**Table 2:** Frequencies and sex differences on items measuring absence and bullying

Response		Total		Girls		Boys	
		*%*	*(N)*	*%*	*(n)*	^*%*^	^*(n)*^
**Absence**							
Truancy	Infrequent	77.7	621,567	77.3	309,094	78.2	312,473
	Moderate	17.9	143,386	18.7	74,809	17.1	68,577
	Frequent	4.3	34,416	4.1	15,874	4.6	18,542
Illness Absence	Infrequent	67.6	557,457	66.3	273,290	68.9	284,167
	Moderate	20.3	167,222	21.5	88,501	19.1	78,721
	Frequent	12.1	99,919	12.3	50,720	11.9	49,199
**Bullying involvement**							
Victimization	Several times a week	3.1	25,872	2.2	9383	3.9	16,489
	Once a week	3.8	31,786	3.3	13,961	4.2	17,825
	Less frequently	24.8	208,672	22.6	95,251	27.0	113,421
	Not at all	68.4	575,872	71.9	302,815	64.9	273,957
Perpetration	Several times a week	2.3	19,261	0.8	3522	3.7	15,739
	Once a week	3.1	26,399	1.6	6760	4.7	19,639
	Less frequently	29.0	244,232	21.1	88,665	37.0	155,567
	Not at all	65.5	551,547	76.6	322,057	54.6	229,490
Victimization-perpetration	Yes	1.6	13,820	0.6	2656	2.7	11,164
	No	98.4	826,452	99.4	417,925	97.3	408,527

Note. Illness absences: infrequent (none and 1 day. and not at all; and a few times per year). moderate (2–3 days and monthly) or frequent (more than 3 days or weekly; and daily or almost daily). Truancy: infrequent (none and not at all) moderate (1 day or 2–3 days; and a few times per year or monthly) and frequent (more than 3 days and weekly; and daily or nearly daily). All gender differences ($\chi$^2^ were significant at level p < 0.001

### Time trends in Illness Absences and Truancy

During the entire study period, 7% of students reported bullying victimization once a week or more often (Table 2). Bullying victimization was more frequent among boys than girls (8% vs. 5%. χ² = 5540.09. p < 0.001; Table 2). Boys also reported bullying perpetration more often than did girls (9% vs. 3% weekly level χ² = 47893.86. p < 0.001). Boys reported bullying perpetration more often than victimization, whereas girls reported more victimization than perpetration. 2% of the study population reported victimization-perpetration, with 3% of boys and less than 1% of girls reporting at least weekly occurrence (χ² = 5343.71. p < 0.001).

**Fig. 1:**
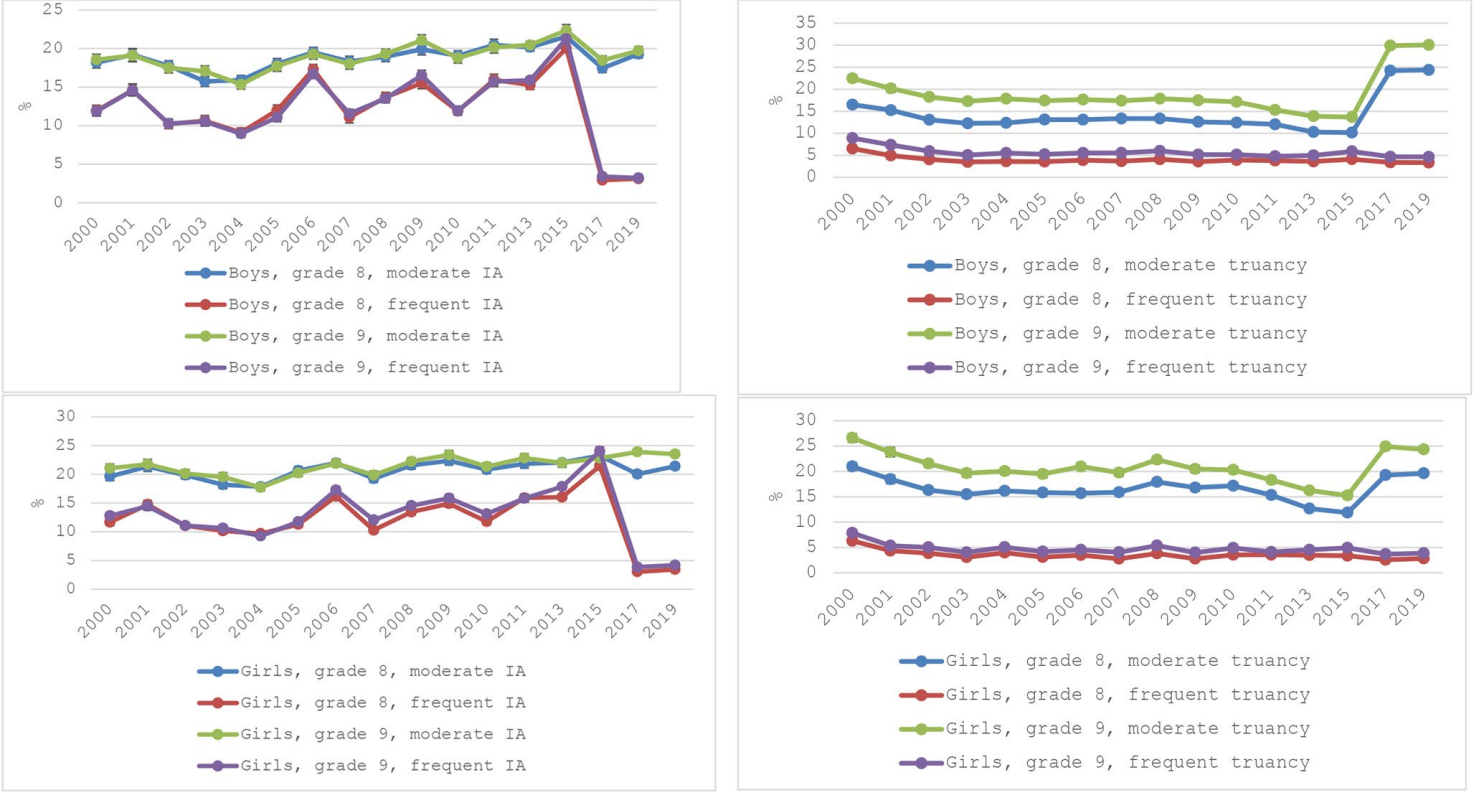
Time trends in absences. Panel A Illness absence separately per sex and grade, and Panel B, truancy separately per sex and grade

Figure 1 depicts the absence rates for each study year. Overall, IA increased between 2000 and 2015, with a pronounced increase in frequent IA for both boys (from 12 to 21%) and girls (from 12 to 23%; Fig. 1, Panel A). In 2017–2019, rates of frequent IA were 3.4 − 4.3%. Truancy rates declined between 2000 and 2015. Frequent truancy declined for all students from approximately 8 to 4%, and moderate truancy for boys (grade 9 from 23 to 13%) and girls (26% to15%; Table 3; Fig. 1, Panel B). In 2017–2019, 27% of boys and 22% of girls reported infrequent truancy.

**Table 3:** Multivariate logistic regression analysis of factors predicting illness absence and truancy. Odds ratios with 95% confidence intervals

		Truancy		Illness absences	
		OR	95% CI	OR	95% CI
Gender	Girl	Ref		Ref	
	Boy	0.72	0.72–0.73	0.83	0.82–0.84
Grade					
	9th grade	Ref		Ref	
	8th grade	0.70	0.69–0.70	0.95	0.94–0.95
Year					
	2000–2001	Ref		Ref	
	2002–2003	0.71	0.69–0.72	0.82	0.80–0.83
	2004–2005	0.70	0.68–0.71	0.79	0.77–0.81
	2006–2007	0.70	0.68–0.71	1.06	1.04–1.08
	2008–2009	0.70	0.69–0.72	1.12	1.09–1.14
	2010–2011	0.66	0.64–0.67	1.06	1.04–1.08
	2012–2013	0.62	0.60–0.63	1.26	1.24–1.29
	2014–2015	0.67	0.65–0.69	1.77	1.73–1.81
	2016–2017	1.25	1.22–1.28	0.60	0.59–0.62
	2018–2019	1.29	1.26–1.32	0.65	0.63–0.66
Bullying victimization					
	Not at all	Ref		Ref	
	Less frequently (than once a week)	0.94	0.88–0.91	1.10	1.09–1.12
	About once a week	0.99	0.97–1.02	1.18	1.15–1.21
	Several times a week	1.45	1.41–1.49	1.46	1.43–1.50
Bullying perpetration					
	Not at all	Ref		Ref	
	Less frequently (than once a week)	2.29	2.26–2.31	1.19	1.18–1.20
	About once a week	4.42	4.31–4.53	1.35	1.31–1.38
	Several times a week	8.75	8.50- 9.00	1.85	1.79–1.90
Bullying victimization-perpetration					
	No	Ref		Ref	
	Several times a week	4.48	4.34–4.62	1.76	1.71–1.81

### Correlations Between Illness Absences, Truancy, and Bullying Over Time

All correlations between both truancy and illness absences and bullying were small, and the highest correlation was found between truancy and bullying perpetration. The range of correlations for all study years were: IA and bullying victimization (range *r*_*spearman*_ = 0.02–0.10), perpetration (range *r*_*spearman*_ = 0.03–0.09) and victimization-perpetration (range *r*_*spearman*_ = 0.02–0.08) and between truancy and bullying victimization(range *r*_*spearman*_ = 0.04–0.12), perpetration (range *r*_*spearman*_ = 0.14–0.19) and victimization-perpetration (range *r*_*spearman*_ = 0.07–0.14). Correlations were slightly higher between truancy and perpetration than between the other variables. The correlation between truancy and victimization tripled over the study period (from 0.04 to 0.12), but was still small in size, when controlling for age, gender and maternal education.

### Results from Multivariate Analyses

In the logistic regression, odds for IA were higher for older students, girls, years 2006–2015, and any type of bullying involvement (Table 3). IA increased steadily from 2000 to 2015, with odds for IA being 77% higher in 2015 compared to 2000. From 2017 to 2019, odds for IA increased slightly.

Older age, being a girl, and any type of bullying involvement were associated with increased odds for truancy. Odds for truancy decreased steadily from 2000 to 2015, with a 33% lower odds for reported truancy in 2015 compared to 2000. From 2017 to 2019, the odds for truancy increased slightly.

Over the complete study period, both bullying victimization and perpetration were associated with both IA and truancy. The odds for IA and truancy increased by 45% if bullying victimization was reported several times a week. Bullying perpetration increased the odds for truancy in a dose-response manner: bullying perpetration less than weekly showed 129% increased odds of truancy (OR = 2.29), weekly occurrence increasing odds to 342% (OR = 4.42), and perpetration several times a week was associated with 775% higher odds for truancy (OR = 8.75). The same dose-response pattern was found for IA, although ORs were lower than for truancy. Also bullying victimization-perpetration was associated with both kinds of absences, with higher odds again found for truancy than for IA.

## Discussion

Our data provides insight into time trends, showing a steady increase of IA and decrease of truancy from 2000 to 2015. In 2017–2019, the rates of IA and truancy were stable, but very different from the prior time series due to the change of item. Bullying victimization, perpetration and victimization-perpetration were associated with both IA and truancy.

During the study period 2000–2015, reported absenteeism changed significantly. IA increased steadily from 2006 to 2007 onwards, with an especially marked increase in the frequent occurrence of IA. In 2015, moderate and frequent IA was reported by 42% of boys and 46% of girls. This is nearly double the percentage reported in the lowest year, 2004, when 24% of boys and 27% of girls reported IA. Compared to international cross-sectional studies utilizing the same “absence during the prior 30-day” question, IA in Finland seems to be somewhat higher than the level reported in the Netherlands, where 33–35% reported IA in the prior month [23]. The increase in self-reported IA during 2000 to 2015 is a significant finding. Health related issues have previously been the most common reasons for absences when reported by students [24], parents, school health care professionals [25], and teachers [26].

The reasons for the increase in IA need to be considered. According to population surveys both internationally and in Finland, an increasing proportion of adolescents report mental health problems, especially depression and anxiety, and prevalence rates show a continuous rise from 1990 to 2020 [27]. Both depressive and anxiety disorders and symptoms have consistently correlated with school absenteeism [26, 27]. Also, due to changes in the cultural climate toward a more open public discussion of mental health topics [31], adolescents may be more prone to disclose their emotional difficulties and symptoms to their parents. Parental attitudes towards illness absences due to these reasons may also have changed. Indeed, “mental health days”, meaning taking a day off from school, are often referred to on social media, or in everyday language of adolescents. As parents/caregivers give permission to these days, both parents and adolescents may consider them as IA. Thus, what once was considered truancy might now be viewed as IA due to a shift in mindset.

In 2017–2019, 3–4% of students reported weekly IA and 18–24% of adolescents reported monthly IA. What appears to be a decline compared to the prior years is likely explained by the change in wording of the item. Also, weekly absences are understandably less common compared with more than 3 days in a month, since the latter may also capture consecutive absence days, for example due to a cold.

The truancy rate found in the current study is in line with that reported in other studies: 13% in Finland reported by the OECD [8], with a 23% decline in self-reported truancy in Finland between 2015 and 2018 [8]. The present data also shows a declining trend in truancy levels during the study period. This decline may partly be explained by the implementation of anti-bullying programs, which have been widely implemented from 2007 onwards [30]. Other preventive programs aiming to increase wellbeing and a new curriculum based on positive pedagogy has also been implemented during the study period [31]. The use of electronic student management systems to track absences has increased nationally in Finland during the study period, with both parents and teachers reporting absences in the same system. Furthermore, the prevalence of behavior problems among 9th graders in Finland has decreased between 2000 and 2019, [27], possibly also reflected in declining truancy levels.

Bullying victimization and perpetration were related to increased odds for IA. As bullying involvement has been associated with somatic and internalizing symptoms, [32] our results may also reflect the adverse psychological effects of bullying involvement.

Consistent with results from several earlier studies showing associations between truancy and conduct disorders in adolescents [reviewed in 33], our regression analysis showed that students who reported bullying perpetration had a notably heightened risk for truancy. Victim-perpetrators also showed a marked increase in the odds for reporting truancy. In addition, results showed an increase, albeit small, over years in the correlation between victimization and truancy, when controlling for covariates such as gender, grade and mother’s educational level. The practical implication of these findings for schools could include arranging holistic assessment and support for students who bully others or regularly skip school, because they may display disruptive behaviors related to peers and authorities alike [34].

The strengths of the current study were the large, nationally representative cohorts and recurring items in the survey. The School Health Promotion study reaches a large proportion of Finnish adolescents. Adolescents with excessive absences may, however, have been absent from school on the survey day, which may lead to an underestimation of absenteeism. The change of wording in the item on the frequency of absences between 2000 and 2015 and 2017–2019 impeded some analyses. Furthermore, it may be difficult for the adolescent to recall events retrospectively for the entire year, thus increasing the likelihood of recall bias in the last two years. This has been noted also in prior studies, and Keppens et al. (2019) reminded that the longer the time frame for self-report, the greater the deviation from actual absenteeism rate [9]. In addition, the item measuring bullying involvement did not include an example on cyberbullying and thus the responses plausibly reflected only traditional bullying.

In conclusion, we found significant temporal changes in both IA and truancy in Finland between 2000 and 2019. Rates of IA increased during most of the study period, whereas truancy rates showed a concurrent decline. However, a relatively stable 3–4% of students reported frequent truancy. Both IA and truancy were associated with bullying involvement. A follow up on absenteeism during and after the COVID-19 pandemic would be important due to increased mental health problems as well as a change in the thinking about when IA is needed.

## Summary

This study examined how the rate of school non-attendance due to illness absences and truancy have changed between 2000 and 2019. We also examined how absences were related to bullying involvement, as either victimization, perpetration or victimization-perpetration. We used a large nationwide dataset of 13-17-year-old Finnish middle school students, who participated in the biennial School Health Promotion study. Frequent IA increased from 12% to 2000 to 22% in 2015. In 2017–2019, frequent IA was reported by 3.5%. Frequent truancy declined from 9% to 2000 to 4% in 2015, and remained stable at 4% in 2017–2019. Bullying victimization was reported at least weekly by 6.9%, perpetration by 5.4% and victimization-perpetration by 1.9% of adolescents. In a logistic regression model, all kinds of bullying involvement increased odds for both IA and truancy. Since bullying involvement was associated with both IA and truancy, particular concern should be raised for adolescents involved in bullying, and for their social and educational functioning. The concurrent increase in IA and decrease in truancy may reflect destigmatization of mental health problems or other changing patterns in reporting absenteeism.

## Data Availability

Data was obtained from the Institute of Health and Welfare, https://findata.fi/en/.
