# Correction to: Time Trends in Adolescent School Absences and Associated Bullying Involvement Between 2000 and 2019: A Nationwide Study

**DOI:** 10.1007/s10578-023-01604-y

**Published:** 2023-09-09

**Authors:** K. Alanko, K. Melander, K. Ranta, J. Engblom, S. Kosola

**Affiliations:** 1 Faculty of Humanities, Psychology and Theology, Åbo Akademi University, Turku, Finland; 2 Tampere University Hospital, and University of Tampere, University of Helsinki, Helsinki, Finland; 3 Faculty of Social Sciences, Faculty of Medicine, Tampere University, University of Helsinki, Tampere, Helsinki, Finland; 4 Department of Mathematics and Statistics, School of Economics, University of Turku, University of Turku, Turku, Finland; 5 Pediatric Research Center, New Children’s Hospital, Helsinki University Hospital, University of Helsinki, Helsinki, Finland

**Correction to: Child Psychiatry & Human Development** 10.1007/s10578-023-01601-1

In the original article, unfortunately Dr. Katarina Alanko's name was incorrectly displayed. Instead of K. Alanko it has been wrongly displayed as E. G. Katarina Alanko. This has been corrected by publishing this correction article. The original article has been corrected.

